# Reading from a Head-Fixed Display during Walking: Adverse Effects of Gaze Stabilization Mechanisms

**DOI:** 10.1371/journal.pone.0129902

**Published:** 2015-06-08

**Authors:** Olivier Borg, Remy Casanova, Reinoud J. Bootsma

**Affiliations:** 1 Institut des Sciences du Mouvement, Aix-Marseille Université, CNRS, Marseille, France; 2 Oxylane R&D, Villeneuve d’Ascq, France; University of Leicester, UNITED KINGDOM

## Abstract

Reading performance during standing and walking was assessed for information presented on earth-fixed and head-fixed displays by determining the minimal duration during which a numerical time stimulus needed to be presented for 50% correct naming answers. Reading from the earth-fixed display was comparable during standing and walking, with optimal performance being attained for visual character sizes in the range of 0.2° to 1°. Reading from the head-fixed display was impaired for small (0.2-0.3°) and large (5°) visual character sizes, especially during walking. Analysis of head and eye movements demonstrated that retinal slip was larger during walking than during standing, but remained within the functional acuity range when reading from the earth-fixed display. The detrimental effects on performance of reading from the head-fixed display during walking could be attributed to loss of acuity resulting from large retinal slip. Because walking activated the angular vestibulo-ocular reflex, the resulting compensatory eye movements acted to stabilize gaze on the information presented on the earth-fixed display but destabilized gaze from the information presented on the head-fixed display. We conclude that the gaze stabilization mechanisms that normally allow visual performance to be maintained during physical activity adversely affect reading performance when the information is presented on a display attached to the head.

## Introduction

The photosensitive area of the human eye (retina) only has a small central region (fovea) on which a visual target needs to be stabilized for tasks requiring high-acuity discrimination. Movements of the head (and consequently the eyes) engendered by natural activities such as walking thus need to be compensated for in order to maintain visual performance while moving around in the environment. Several mechanisms for such gaze stabilization have been described, compensating for the translational and rotational head movement characteristics of walking [1–6]. These gaze stabilization mechanisms operate at two complementary functional levels: while movement of the head is accompanied by compensatory eye movements, primarily originating from the vestibulo-ocular reflexes (VOR), the head itself is stabilized in space by means of the vestibulo-collic reflexes (VCR).

Walking gives rise to vertical and horizontal translational movements of the head at, respectively, step and stride frequencies. For an average-length person walking at a speed of 6 km/h (i.e., 1.67 m/s), this corresponds to approximately 2-Hz vertical head movements and 1-Hz horizontal head movements [4,5,7]. Such translational head movements activate the linear VCR (lVCR) that generates compensatory head rotations in the pitch and yaw directions [4,5]. A similar mechanism, the angular VCR (aVCR), operates to compensate the head rotations resulting from trunk rotation during walking [8,9]. Together, these vestibulo-collic reflexes tend to maintain the naso-occipital axis of the head oriented toward a point in space known as the head fixation point [4,5,10]. As walking speed increases, the location of the head fixation point moves forward, stabilizing at around 0.8 m in front of the participant for walking speeds of 1.2 m/s and higher [8]. The VCR-based stabilization of head orientation is complemented with VOR-based compensatory eye movements in order to stabilize the gaze on the visual target. The short-latency responses of the linear and angular vestibulo-ocular reflexes (<15 ms [11,12]) can be supplemented by visually mediated smooth pursuit or optokinetic eye movements occurring at longer latencies (~100ms [13–17]). These latter delays, inherent in visual signal processing, may be cancelled by predictive mechanisms [18–20]. Fixation distance also plays an important role in the VORs response amplitude modulation [4–6,21–26], as well as in the VCR response modulation [4,5]. For the present purposes, we refer to the contributions of all these factors to compensatory eye rotations as the enhanced VOR (i.e., EVOR [20]).

During walking the retinal image of an earth-fixed target located beyond the head fixation point is quite effectively stabilized by an EVOR gain—the ratio of eye to head angular velocity—close to 1 and an EVOR phase—the asynchrony between eye and head angular velocity signals—around 180° [1,2,4–6]. In these and indeed the grand majority of studies on gaze stabilization image stability is operationally defined by the degree of motion of the image across the retina (i.e., retinal slip). We know of only one study in which the effect of walking on visual performance was directly tested: [3] reported that visual acuity was largely, although not completely, preserved. One of the goals of the present study is therefore to provide a functional evaluation of visual performance during walking. Its main goal however is to compare performance during walking when reading information from an earth-fixed display and from a head-fixed display. Recent technological advances have allowed the development of lightweight head-up displays that can be worn during daily activities, but the potential effects of natural activities on reading from such head-up displays have not yet been sufficiently evaluated.

During walking head rotations present the same characteristics when viewing either an earth-fixed or a head-fixed target [27]. It is therefore likely that they elicit similar VOR responses. The VOR has indeed been reported to be active when participants viewing a head-fixed target are moved passively at frequencies or velocities that fall within the range observed during walking [6,14,20]. However, since a head-fixed visual target moves in tandem with the head, properly fixating the target in fact requires that the eyes do not move with respect to the head. Thus, during walking EVOR-induced eye rotations would generate, rather than eliminate, retinal slip and thus impede on visual performance. Visual acuity has been reported to decrease rapidly when retinal slip exceeds a threshold of approximately 6 deg/s [28,29]. While gaze stabilization mechanisms maintain retinal slip below this threshold when viewing an earth-fixed target at far distance during walking [1,3,4], the same mechanisms may be expected to generate retinal slip above this threshold when viewing a head-fixed target under the same conditions.

In the present study, we assessed visual performance when reading information from an earth-fixed display and from a head-fixed display while participants were either standing or walking. To this end we determined in each of the four experimental conditions the minimal duration over which a stimulus needs to be presented to allow accurate reading of numerical time information that could be presented at different visual sizes. For seated participants optimal reading performance (that is, smallest minimal stimulus presentation durations) was attained for visual character sizes in the range of 0.2–1.0 degrees [30]. Because smaller characters require higher visual acuity for reading, we expected the perceptual threshold to increase for the smaller character sizes when reading from a head-fixed display during walking as compared to the other three conditions. Using motion analysis of the head and eyes, we complemented the functional assessment of visual performance with measures of retinal slip and characteristics of the operative EVOR.

## Materials and Methods

### Participants

Fourteen young healthy adults (7 men and 7 women, age 25 ± 3.0 years) participated in the study. All had a corrected or uncorrected visual acuity of at least 10/10 for each eye, as determined by a 5-m Monoyer test. None reported a history of disease or disorder that would affect performance under the experimental conditions. Participants provided written consent prior to participation. The study was approved by the local institutional review board of the Institute of Movement Sciences (*Comité Ethique de l’Institut des Sciences du Mouvement d’Aix-Marseille Université*) and conducted according to University regulations and the 1964 Declaration of Helsinki.

### Task and procedure

The participants’ task was to read visually-presented numerical time information out loud. Stimuli were presented on an earth-fixed display (EFD) or on a head-fixed display (HFD), while participants stood or walked on a treadmill. All participants performed the reading task under the four conditions resulting from the combination of two displays (EFD and HFD) and two activities (Stand and Walk).

Under the EFD conditions, stimuli were presented in the center of a 55-inch LCD screen (Samsung ED55C, 1920 x 1080 pixel resolution) fixed to the wall of the experimental room at a distance of 2.0 m in front of the participant. The center of the screen was aligned with the participant’s standing eye level. Under the HFD conditions, participants were equipped with a head-mounted personal viewer (Sony HMZ-T2 with 2 OLED screens, 1280 x 720 pixel resolution, 0.3-kg weight) and stimuli were presented in the center of a virtual 75-inch screen positioned at a virtual distance of 2 m. The center of the screen was aligned with the standing participant’s naso-occipital axis. Both screens operated at 60 Hz. Synchronization with the 16.67-ms refreshment cycle of the screens was controlled by E-Prime 2.0 software (Psychology Software Tools, Inc., USA), running on a HP Z400 Workstation (Intel Xeon CPU W3520 @ 2.67GHz 1.57GHz, 3 Go RAM, NVIDIA Quadro FX 1800 graphic card, Microsoft Windows XP Professional SP3 OS).

**Fig 1:**
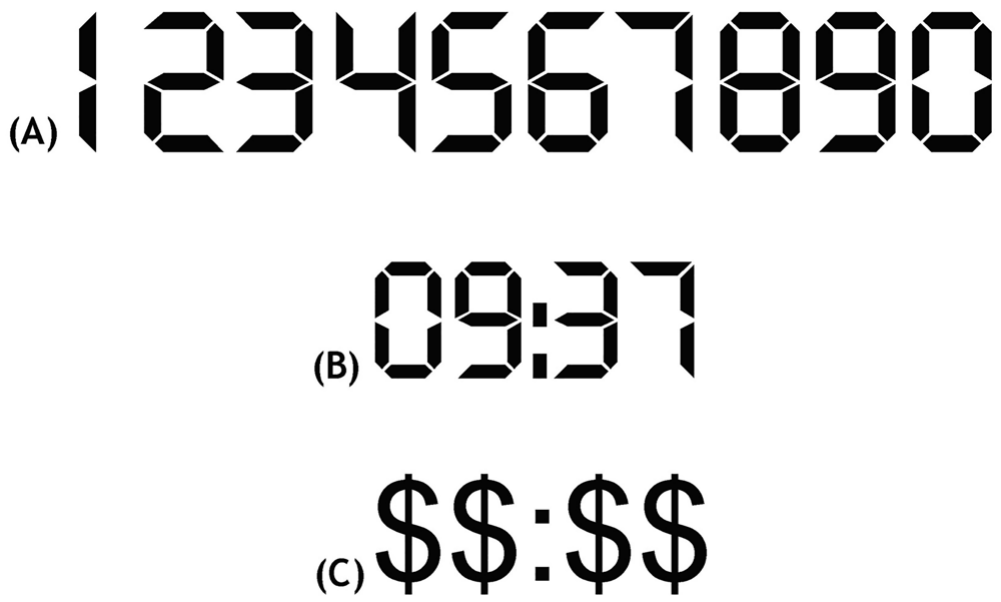
Stimulus presentation material. (A) Numerical characters of the Digiface font; (B), a stimulus example; (C), the 500-ms mask.

Stimuli consisted of two two-digit numbers, each randomly selected from the pool of 60 possibilities, representing time information in a numerical format (“mm:ss” with 00≤mm≤59; 00≤ss≤59). Each stimulus presentation was immediately followed by a 500-ms mask (“$:$”). Characters were presented in the Digiface font (see Fig 1), a constant-width font comparable to the 7-segment fonts used in classical LCD displays. They were presented in mesopic viewing conditions, in black against a white 100 cd/m^2^-luminance background with a mean Michelson contrast of 80% and an image resolution of more than 30 pixels per degree of visual angle on both displays.

On each trial, a fixation cross was presented in the center of the screen during 2 s, followed by a stimulus (presented for a duration depending on the staircase procedure to be described) and the 500-ms mask. Before the start of the experimental phase, participants were familiarized with the reading task during a one-minute training session with fixed presentation durations. During the experimental phase, after each stimulus presentation participants verbalized the perceived sequence of numbers that was immediately entered on a keyboard by the experimenter. The inter-stimulus interval was about 5 s.

Stimulus presentation duration was adapted during the sequence using an adaptive one-down/one-up staircase, leading to the threshold for 50% correct responses [31]. Starting from a sufficiently long initial stimulus presentation duration, this duration was decreased in time steps of 66.67 ms (four times the duration of the refreshment cycle of the screen) following each correct response; when an error occurred, presentation duration was increased by 66.67 ms. After the first four inversions, the step size was reduced to 33.33 ms. After the next three inversions, the step size was further reduced to 16.67 ms. The procedure ended after 12 inversions. The perceptual threshold (corresponding to the stimulus presentation duration for 50% correct responses) was calculated as the mean of the last four inversion values.

Perceptual thresholds were obtained in each of the four testing conditions (EFD-Stand, EFD-Walk, HFD-Stand et HFD-Walk) for each of six different visual character sizes (0.2°, 0.3°, 0.5°, 1.0°, 2.0°, and 5.0°, corresponding to character width). Thus, participants performed a total of four blocks of trials (one under each testing condition), with each block presenting six interlaced (visual size) staircase procedures for the same testing condition. Each block took about 15 min. Blocks were presented in a randomized order with a minimum of 5 min of rest between blocks. All tests were performed in binocular viewing conditions. Each participant completed the full experimental session on a single day in order to minimize within-participant variability.

Wearing sports shoes participants either stood or walked on a motor-driven treadmill (Sprint, Medical Development, Tecmachine HEF, France) equipped with a 0.8-m wide belt. In the standing condition, the treadmill belt did not move. In the Walking conditions the treadmill belt speed was adjusted (1.2 ± 0.05 m/s) so that each participant adopted a stable stepping frequency of 1.8 Hz (close to the preferred stepping frequency for moderate walking speed [8,32]. This stepping frequency was expected to induce vertical head motion at 1.8 Hz and horizontal head motion at 0.9 Hz.

In both Standing and Walking conditions, longitudinal position on the treadmill (and therefore viewing distance in EFD conditions) was maintained by means of a flexible strap fastened around the participant’s waist. Participants were asked to slightly tend the strap throughout the tests and try and remain near the longitudinal axis of the treadmill. The HFD allowed sufficient peripheral vision in the lower visual field to maintain a correct lateral position [33,34].

### Motion capture

In order to capture head motion during the experimental sessions participants wore a modified cycling helmet, firmly attached to the head and equipped with four 5-g retroreflective markers (total weight < 0.2 kg). Three others markers permitted capturing trunk motion: one on each acromioclavicular joint and one on the jugular notch where the clavicles meet the sternum. The seven markers were tracked using a five-camera VICON 624 motion analysis system (VICON Motion System, Lake Forest, CA) operating at 120 Hz. The system was recalibrated every day.

A calibration procedure run prior to the experimental session allowed the position of the head to be determined from the positions of the markers on the helmet. During this calibration procedure, the participant stood on the treadmill while wearing the helmet together with two additional head markers: one on the occipital protuberance and one on the nasal bone. The 3D positions of all these six markers, which defined head position and the orientation of the naso-occipital axis with respect to the helmet, were recorded over a period of 10 s.

Eye movements were recorded using a BIOPAC MP150 BioNomadix wireless electro-oculography (EOG) system (Biopac Systems, Inc., Santa Barbara, CA) operating at 120 Hz. With a ground electrode placed on the head in the middle of the frontal bone, horizontal eye movement was captured via two EOG electrodes placed on the outer canthus of the left and right eyes. Two others electrodes were placed above and below the dominant eye to capture vertical eye movements.

EOG voltages obtained during the experimental sessions were transformed into horizontal and vertical eye positions on the basis of a second calibration procedure performed before the experimental sessions. During this calibration procedure seated participants immobilized their head in a chin rest. Without blinking they fixated 1° visual targets appearing in front of them at ±8° and ±16° horizontal and ±5° and ±10° vertical eccentricities. EOG voltages recorded during the first 50 ms after the end of the saccade to the target were averaged to minimize the baseline drift effect occurring during fixation.

The time lines of stimulus presentation and eye movement were synchronized with the VICON recording of head and trunk movement by inputting the analog signals (from E-Prime and EOG recordings) into the VICON recording system via a 64-channel Mezzanine card (VICON Motion System, Lake Forest, CA).

### Data processing

The position time series of eye, head, and trunk were filtered using a fifth-order Butterworth 0.1–6 Hz band-pass filter. The lower cutoff effectively removed baseline drift notably present in the EOG signal, while the higher cutoff was based on previous work on locomotion [4,8,35]. As expected fast Fourier transformations of the velocity signals obtained by differentiation of the position time series revealed predominant frequencies [4,8,36] at 1.8 Hz for vertical movements and at 0.9 Hz for horizontal movements.

**Fig 2:**
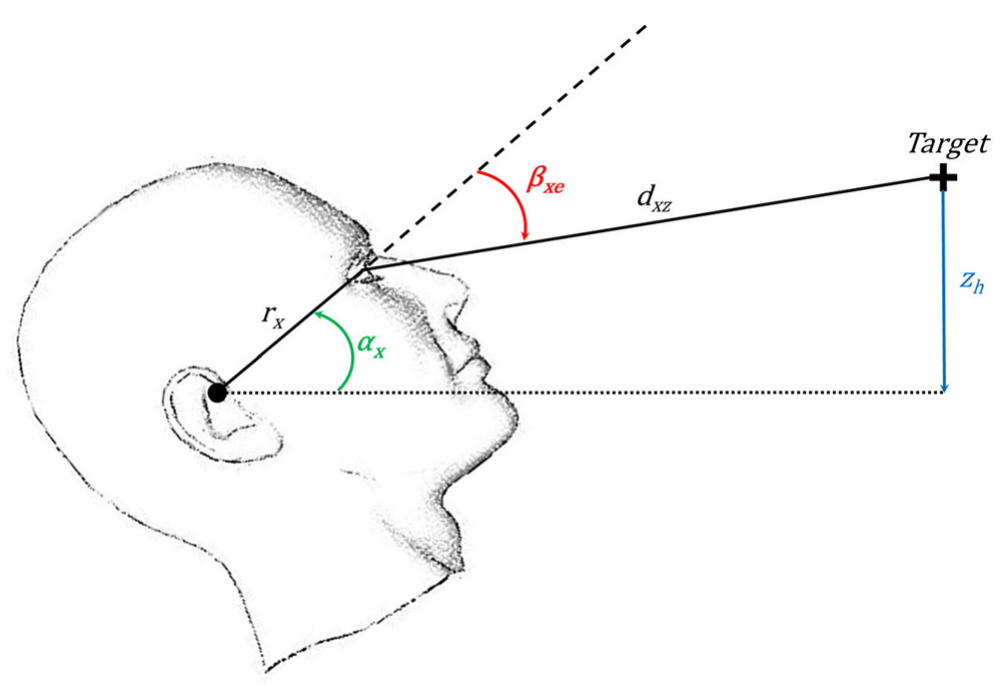
Method used to calculate ideal eye pitch *β*_*xe*_ (see [2,27] for details and discussion). *z*_*h*_: vertical distance between the head rotation axis (black circle) and the target (black +), *α*_*x*_: head pitch, *r*_*x*_: distance between head rotation axis and eye rotation axis, *d*_*xz*_: distance from the eye to the target.

Motion analysis was restricted to the epochs in which participants had to focus their attention on the reading task as signaled by the appearance of the fixation cross. Operationally defined as the 1.7-s period preceding the end of each stimulus presentation, these epochs typically contained three steps in the walking conditions. To further minimize the EOG baseline drift effect, the first five epochs without eye blinking recorded during the first minute of a trial were selected for analysis. Because the reading task involved stimuli of considerable horizontal extent (Fig 1), analyses of retinal slip and VOR were limited to the vertical direction / sagittal plane. Median retinal slip speed was calculated from the difference between the ideal and measured eye angular velocity signals according to the method of [2,27]. See Fig 2 for details.

$$\beta_{xe}\;={\text{ sin}}^{-1}\left(\frac{z_{h}\left(t\right)-r_{x}\cdot \text{sin }\alpha_{x}\left(t\right)}{d_{xz}\left(t\right)}\right)-\alpha_{x}\left(t\right)$$

The magnitude of the first peak of the cross-correlation function was used to determine the strength of the relationship between signals of interest while the corresponding time lag informed about the temporal offset of the two signals. The predominant frequency of each signal—estimated from the peak of the power spectrum—was used to determine the signal period. The temporal lag was then divided by the period and multiplied by 360 to obtain the phase relationship in degrees between the two signals. For negative cross-correlation peaks indicating antiphase behavior, we added 180° to the calculated phase shifts. EVOR gain was obtained by calculating the median ratio of (the absolute values of) eye rotation velocity over head rotation velocity. In the same way, EVOR sensitivity was obtained by calculating the median ratio of (the absolute values of) head translation velocity over eye rotation velocity. Phase shift as well as EVOR gain and sensitivity were only considered reliable when the absolute value of the peak cross-correlation value was ≥ .3.

### Statistical analysis

After checking for outliers and normality of the data distribution with boxplot and Shapiro-Wilk tests, perceptual thresholds for reading were evaluated by a three-way repeated-measures Analysis of Variance (ANOVA), with Display Type (EFD and HFD), Activity (Standing and Walking) and Visual Size as within-subjects factors. Two-way repeated-measures ANOVA were performed to determine the effect of Display Type and Activity on Retinal Slip in the sagittal plane. Where appropriate, statistically significant (α = .05) differences were further explored using Tukey HSD post-hoc tests. Fisher z-transformation was applied to correlation coefficients in order to allow Student t-tests for comparing conditions. The means were inverse-transformed for reporting. ANOVA effect sizes were determined using partial *η* ^2^. Data are expressed as mean ± standard deviation, unless otherwise stated. Peak absolute cross-correlation coefficients were considered as high when larger than .5, moderate between .3 and .5, and low when smaller than .3.

## Results and Discussion

Two participants (a 25-years old male and a 20-years old female) could not complete the experiment because they became unwell (see [37]) when wearing the personal viewing system. One other participant had to be excluded due to a hardware failure at the end of the experiment. Analysis thus concerned the full data from 11 participants.

### Minimal stimulus presentation duration

**Fig 3:**
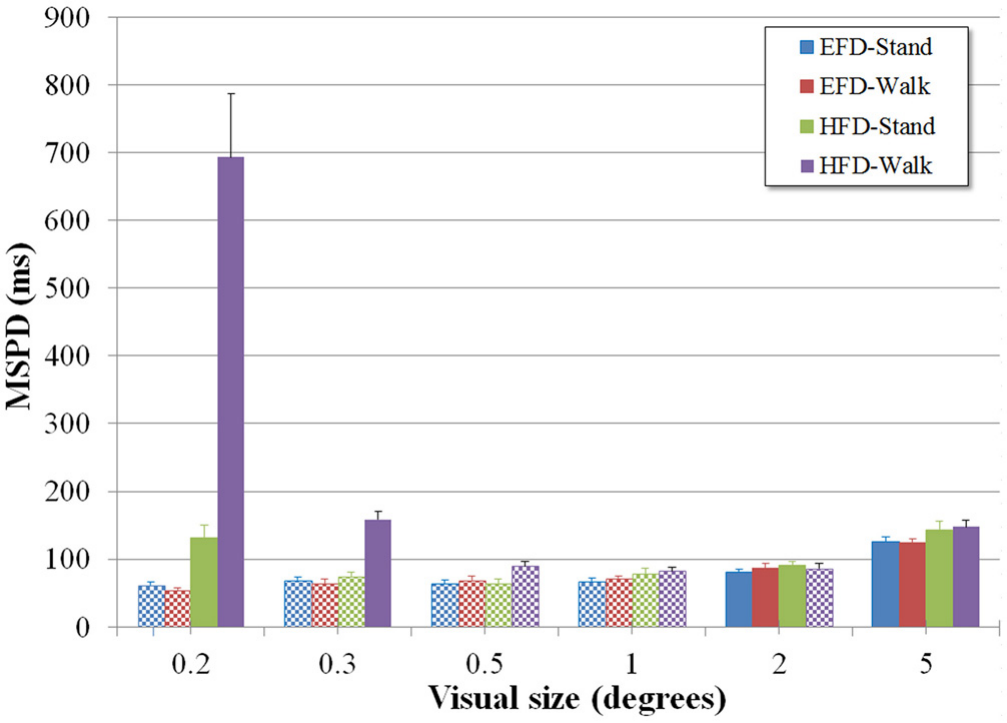
Minimal stimulus presentation duration (MSPD) as a function of visual size for the EFD-Stand, EFD-Walk, HFD-Stand and HFD-Walk conditions. Error bars represent standard error. For each condition, the range of sizes revealing minimal duration thresholds is indicated by chequered textures.

Reading performance was evaluated by the minimal duration during which a stimulus had to be presented to allow (50% correct) reading. Fig 3 presents the between-participant averages of this Minimal Stimulus Presentation Duration (MSPD) under each of the four experimental conditions (EFD-Stand, EFD-Walk, HFD-Stand, HFD-Walk) for the six visual sizes tested (also see S1 File).

The three-way repeated-measures ANOVA on MSPD revealed a complex pattern of results. The main effects of all three factors—Display Type: *F*(1, 10) = 52.3, *p* < .001, *η* ^2^_p_ = .84, Activity: *F*(1, 10) = 48.8, *p* < .001, *η* ^2^_p_ = .83, and Visual Size: *F*(1.14, 11.4) = 31.9, *p* < .001, *η* ^2^_p_ = .76—were qualified by first-order interactions—Display Type x Activity; *F*(1, 10) = 44.2, *p* < .001, *η* ^2^_p_ = .82, Display Type x Visual Size: *F*(1.09, 10.9) = 47.5, *p* < .001, *η* ^2^_p_ = .83, and Activity x Visual Size: *F*(1.08, 10.8) = 36.2, *p* < .001, *η* ^2^_p_ = .78—as well as a second-order interaction Display Type x Activity x Visual Size: *F*(1.07, 10.7) = 37.3, *p* < .001, *η* ^2^_p_ = .79. Post-hoc analysis of the overarching interaction revealed the following results.

As can be seen from Fig 3, the participant’s activity (i.e., standing or walking) did not affect reading performance when the information was presented on the earth-fixed display. For both EFD activities perceptual thresholds were relatively low and stable (i.e., no significant differences) for visual sizes between 0.2° and 1°. MSPD increased when visual size increased from 1° to 2° (*p*s < .05) and from 2° to 5° (*p*s < .05) in both the EFD-Stand and EFD-Walk conditions. In summary, EFD reading performance was affected by visual size at the high end of the range explored (i.e., ≥ 2°), but not by participant activity. The increase in MSPD observed for the larger visual sizes of the present study corresponded to the findings of [30] who used a similar procedure to determine perceptual thresholds when seated participants read information from an earth-fixed display. They attributed this detrimental effect of larger visual sizes to decreasing character acuity in peripheral vision, crowding between adjacent characters, and decreasing accuracy of position signals in peripheral vision [38]. As found here for standing participants, [30] also reported a plateau of optimal performance for a range of visual sizes between 0.2 and 1°. The resolution of the HFD used in the present experiment did not allow to correctly present the information at visual sizes smaller than 0.2°, as this resulted in ragged characters. Thus, the present experimental design did not allow us to verify whether in the earth-fixed display conditions MSPD also increased when visual size approached the lower limits of visual acuity (i.e., at 0.1°), as reported by [30]. Overall, we conclude that reading from an earth-fixed display is comparable when participants are sitting ([30]), standing or walking (present study).

A different picture emerged when participants read the information from the head-fixed display. In the HFD conditions, perceptual thresholds were as low and stable as when reading from the EFD for visual sizes of, respectively, 0.3° to 1° for HFD-Stand and 0.5° to 1° for HFD-Walk (see Fig 3). As in the EFD conditions, MSPD increased for larger visual sizes (i.e., from 1° to 2°, *p* < .05 for HFD-Stand, *ns* for HFD-Walk, and from 2° to 5°, *p*s < .05 for both HFD-Stand and HFD-Walk). Thus, for moderate visual sizes, reading performance was similar in the HFD and EFD conditions for both the standing and walking activities. However, this was not the case for smaller and larger visual sizes. In the HFD-Stand condition reading performance decreased (i.e., MSPD increased, *p* < .01) when the information to be read was presented at the smallest (i.e., 0.2°) visual size. Walking severely affected reading from the HFD for the smaller visual sizes: MSPD increased considerably when the information was presented at a visual size of 0.3° (different from 0.5° and 1°, *p*s < .01); it increased dramatically when the information was presented at a visual size of 0.2° (*p*s < .001). Finally, at the largest visual size (i.e., 5.0°), reading performance decreased significantly in the HFD conditions compared to the EFD conditions, for both standing and walking (*p*s < .05). In summary, compared to EFD conditions, HFD reading performance was more affected by visual size at both the low end and the high end of the range of visual sizes explored (0.2°-5°). The effect of smaller visual sizes in the HFD conditions was much stronger when participants were walking as compared to standing. Because a decrease in reading performance for smaller and larger visual sizes is generally attributed to limitations in visual acuity in central [30,39–41] and peripheral vision [38], respectively, the present results suggested that visual acuity would be reduced when reading from a HFD, especially in central vision during walking.

### Retinal slip

In order to determine whether the above-described effects of walking on reading from a head-fixed display could be indeed attributed to a loss of visual acuity engendered by a difficulty in retinal image stabilization, we analyzed retinal slip in the sagittal plane in each of the four experimental conditions (EFD-Stand, EFD-Walk, HFD-Stand, HFD-Walk).

**Fig 4:**
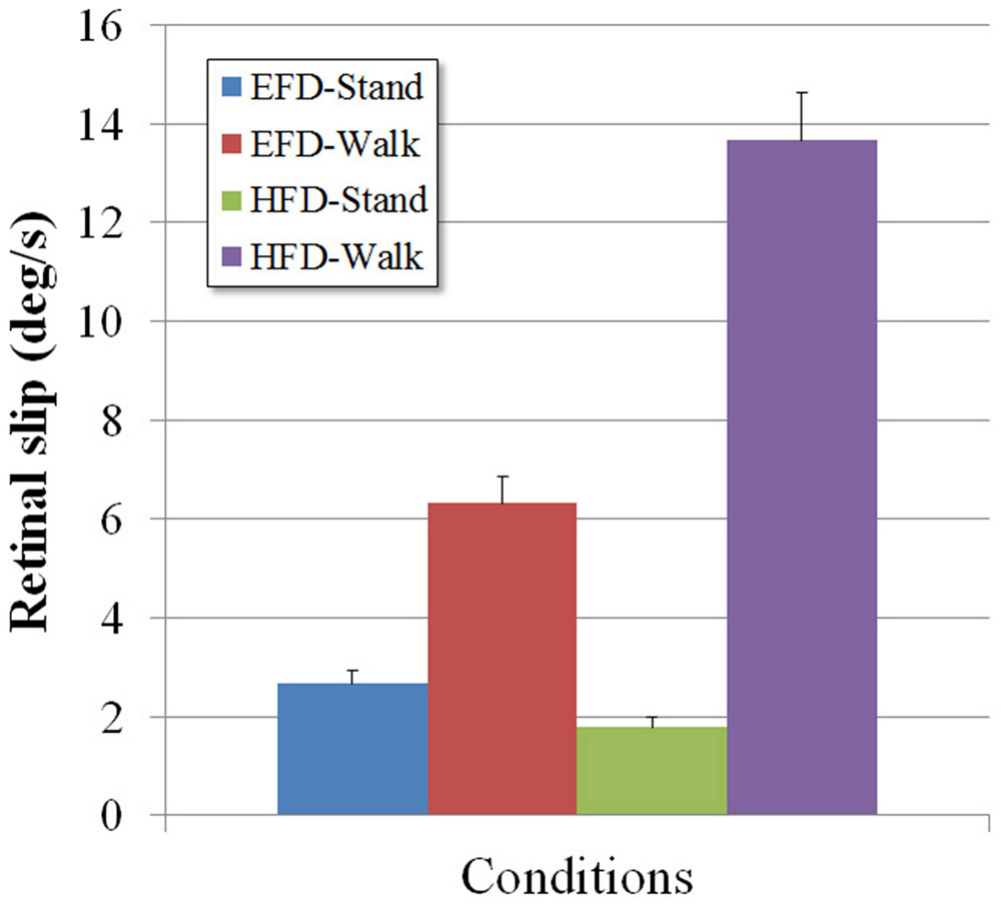
Retinal slip observed in the EFD-Stand, EFD-Walk, HFD-Stand, and HFD-Walk conditions. Error bars represent standard error.

A two-way repeated-measures ANOVA on median retinal slip revealed significant main effects of Display Type (*F*(1, 10) = 37.2, *p* < .001, *η* ^2^_p_ = .79) and Activity (*F*(1, 10) = 186.0, *p* < .001, *η* ^2^_p_ = .95) as well as a significant Display Type x Activity interaction (*F*(1, 10) = 47.4, *p* < .001, *η* ^2^_p_ = .83). Post-hoc analysis of this interaction brought out several interesting effects (see Fig 4).

When the information was presented on the EFD, standing gave rise to a median retinal slip of 2.7 ± 0.9 deg/s; during walking this increased to 6.3 ± 1.8 deg/s. The latter value closely correspond to the 6.8 ± 1.2 deg/s reported by [4] for conditions of comparable walking velocity and visual target distance. Given that EFD reading performance was similar during standing and walking (see Fig 3), we conclude that in both activities the amount of retinal slip remained within the boundaries of optimal visual performance. This corresponds to the threshold of around 6 deg/s reported in earlier studies [28,29].

When the information was presented on the HFD, standing gave rise to a median retinal slip of 1.8 ± 0.7 deg/s; during walking this increased to 13.7 ± 3.2 deg/s. With retinal slip in the HFD-Walk condition clearly exceeding the upper boundary of optimal visual performance, visual acuity must have declined in this condition. As demonstrated by the considerable decrease (Fig 3) in HFD-Walk reading performance for the smaller visual sizes (0.2° and 0.3°), this was indeed the case.

A somewhat unexpected result was that retinal slip was smaller (*p* < .05) under the HFD-Stand condition than under the EFD-Stand condition (Fig 4). Combined with the finding of a simultaneous decrease in reading performance for the smallest (i.e., 2°) visual size (Fig 3), this pattern of results suggests that in the HFD-Stand condition retinal slip may have been too small to allow optimal reading performance. Note that the method of calculating retinal slip, based on the (absolute) difference between the ideal and measured eye angular velocity signals, tends to provide an overestimation: any experimental error in either signal will increase the retinal slip measure. Operational retinal slip was thus probably less than the average 1.8 deg/s reported. The admittedly speculative explanation of insufficient retinal slip for the observed decrease in reading performance is consistent with previous studies showing that when, under laboratory conditions, images are too perfectly stabilized on the retina, perception fades [42–45] due to neural adaptations [46,47], even with brief periods of stabilization [48]. In fact, under natural conditions, even during attempted eye fixation, the eyes move continually with three types of eye movements: microsaccades, drifts, and tremor [43,49,50]. These so called “fixational eye movements” have been linked to the visual restoration of fading perception [43,51,52]. Besides, [53] have shown that fixational eye movements enhance discrimination of high-frequency gratings, suggesting that the physiological retinal slip from these eye movements contributes to the perception of fine spatial detail. [54] already reported that normal retinal slip generated by fixational eye movements prevent perception fading especially for the finest lines they tested, and they further demonstrated that a somewhat exaggerated retinal slip is more effective. This led us think that the pattern of results in the HFD-Stand condition—smaller retinal slip and lower reading performance for the smallest size than for EFD-Stand—was due to additional retinal slip generated by the small-amplitude head and body movements occurring in the EFD-Stand condition. It may combine with the physiological retinal slip and help restore visibility, or prevent fading, of fine spatial details. Moreover, this explanation builds on the suggestion by [55] that the goal of the gaze stabilization mechanisms is to produce nonzero retinal slips that are optimal for vision. Clearly, further research is needed to substantiate this explanation.

Our results thus indicated that for both EFD-Stand and EFD-Walk conditions, optimal reading performance (as assessed by MSPD) was allowed by retinal slip rates in the range of 2.7 to 6.3 deg/s. The reduction in visual acuity in central and peripheral vision associated with the decrease in reading performance for, respectively, the smallest and the largest visual sizes in the HFD conditions was due to inappropriate retinal slip. For HFD-Stand reading performance decreased to a certain extent when character size was reduced to 0.2° or augmented to 5.0°, perhaps because of fading resulting from too little retinal slip. For HFD-Walk, reading performance decreased considerably when character size was reduced to 0.3° or augmented to 5.0°, and dramatically when it was reduced to 0.2° because retinal slip became too large, blurring the image.

### Gaze stabilization mechanisms

**Fig 5:**
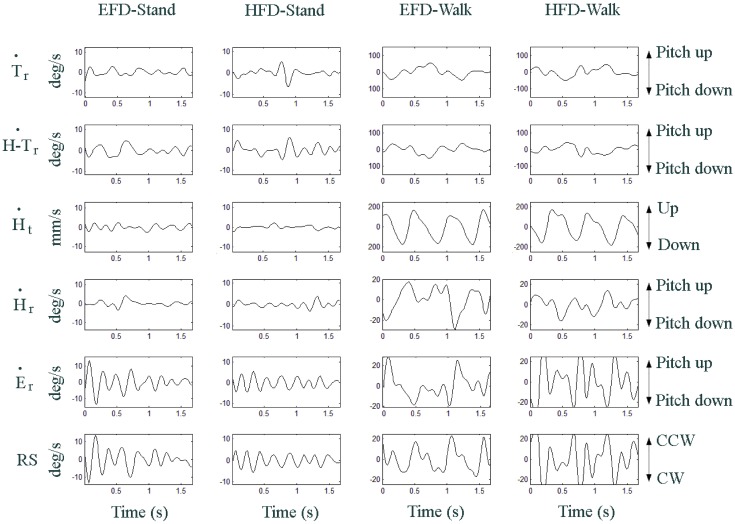
Signal waveform examples under each of the experimental conditions for one participant. From top to bottom: trunk rotation velocity, head-trunk rotation velocity, head translation velocity, head rotation velocity, eye rotation velocity and retinal slip.

Analysis of the linear EVOR involvement in gaze stabilization revealed that peak cross-correlation coefficients between eye rotation velocity and head translation velocity were generally low (*r* < .08) except for EFD-Walk where it was moderate (*r* = .33). This suggested that the linear EVOR did not act to any substantial extent to compensate for head translation in the sagittal plane (also see Fig 5). The present results are thus consistent with earlier studies that also reported no or only weak activation of the lVOR during head translation frequencies of less than 2 Hz while viewing a far target [6,22,23].

Analysis of the angular EVOR involvement in gaze stabilization revealed that peak cross-correlation coefficients between eye rotation and head rotation velocities were quite low for standing conditions (EFD-Stand: *r* = .11; HFD-Stand: *r* = .02). Walking conditions, on the other hand, gave rise to high cross-correlations reaching *r* = .73 for EFD-Walk and *r* = .55 for HFD-Walk.

For EFD-Walk, a 190° ± 13° phase lag between eye rotation velocity and head rotation velocity combined with a 1.08 ± 0.50 EVOR gain indicated that angular EVOR acted to efficiently compensate head rotation (see Fig 5). HFD-Walk demonstrated a 185° ± 37° phase lag and a 2.43 ± 1.38 EVOR gain. The HFD-Walk gain was significantly higher than the EFD-Walk gain (*t*(10) = 3.02, *p* < .05). In order to better understand the origin of this increased gain we performed a spectral analysis of the HFD-Walk condition that revealed common frequencies for eye and head rotation velocities at 1.8 and 3.6 Hz frequencies. Coherence analysis at the 1.8 Hz common eye and head rotation frequency indicated a 165 ± 32° phase lag and a 1.48 ±0.83 EVOR gain (for *r* = .40), suggesting that in the HFD-Walk condition the angular EVOR operated as in the EFD-Walk condition. At the 3.6 Hz common eye and head rotation frequency (observed for HFD-Walk but not EFD-Walk), coherence analysis indicated a 173 ± 24° phase lag and a 5.13 ± 3.42 gain (for *r* = .41). The observed lag of head rotation relative to eye rotation and the presence of the 3.6 Hz common frequency with a reliable gain of more than five suggest that retinal slip induced combined eye-head tracking (CEHT). This eye-head coordination has been shown to produce head movements that typically lag eye movements during target motion tracking [17]—essentially due to large inertia of the head as compared to the eye [56,57]—and smooth pursuit eye movements that typically present eye velocity overshoots and oscillations around the target velocity [15,17,58]. Such CEHT behavior is thus consistent with the pattern of result observed in the HFD-Walk condition.

Overall, our results suggested that during walking the angular EVOR was activated and operated so as to produce eye movements to compensate for head rotation. While this gaze-stabilization mechanism allowed retinal slip to remain within the optimal range for reading from an earth-fixed display, it produced adverse effects in the unnatural condition of reading from a head-fixed display. Here the compensatory eye movements in fact generated retinal slip, leading to a decrease in reading performance for smaller and larger visual character sizes. During standing, head rotation was insufficient to activate the EVOR. The retinal slip generated by the small-amplitude head rotation observed during standing was within the optimal range for reading from the earth-fixed display. With the display fixed to the head retinal slip became very small, perhaps too small, as reading performance was seen to deteriorate at the low end of the range of visual sizes tested.

## Conclusion

The present study demonstrated that under natural viewing conditions (i.e., with the information source anchored to the environment as in the EFD conditions) reading performance was as good during walking as during standing. Optimal performance for reading numerical time information was obtained for visual character sizes in the range of 0.2° to 1°, corresponding to the findings for seated participants reading similar stimuli [30]. Such remarkable reading performance during physical activity was supported by gaze stabilization mechanisms that effectively compensated for the head movements engendered by the walking activity and acted to maintain retinal slip within the functional range, thereby preventing visual acuity from decreasing [1,2,4–6].

When the information source was anchored to the head (as in the HFD conditions), however, the compensatory eye movements resulting from the operation of the same gaze stabilization mechanisms generated rather than eliminated retinal slip during walking. The resulting loss of acuity was evident in the deterioration of reading performance for the larger (5°) but especially the smaller (0.2°-0.3°) visual character sizes. During walking reading from the HFD was thus markedly affected in comparison to reading from the EFD.

## Supporting Information

S1 File Raw MSPD data for each participant in each experimental condition. (XLS)
